# *cis*-Bis[4-amino-*N*-(pyrimidin-2-yl)benzene­sulfonamido-κ^2^ *N*,*N*′]bis­(dimethyl sulfoxide-κ*O*)cadmium

**DOI:** 10.1107/S1600536811010816

**Published:** 2011-03-26

**Authors:** G. M. Golzar Hossain

**Affiliations:** a Department of Chemistry, University of Dhaka, Dhaka 1000, Bangladesh

## Abstract

The complete mol­ecule of the title compound, [Cd(C_10_H_9_N_4_O_2_S)_2_(C_2_H_6_OS)_2_], is completed by the application of a twofold rotation axis. The Cd^II^ atom is six coordinated by two bidentate sulfadiazinate anions and two dimethyl­sulfoxide mol­ecules. The resulting N_4_O_2_ donor set displays a distorted trigonal–prismatic coordination geometry. The S atom and methyl groups of dimethyl­sulfoxide are disordered over two sets of sites, with site occupancies of 0.715 (4) and 0.285 (4). The crystal structure features inter­molecular N—H⋯N and N—H⋯O hydrogen bonds that lead to the formation of layers in the *ab* plane.

## Related literature

For related structures, see: Heren *et al.* (2006 ▶); Hossain & Amoroso (2007 ▶). For background to hydrogen bonds formed by sulfadiazinate anions, see: Paşaoğlu *et al.* (2008 ▶).
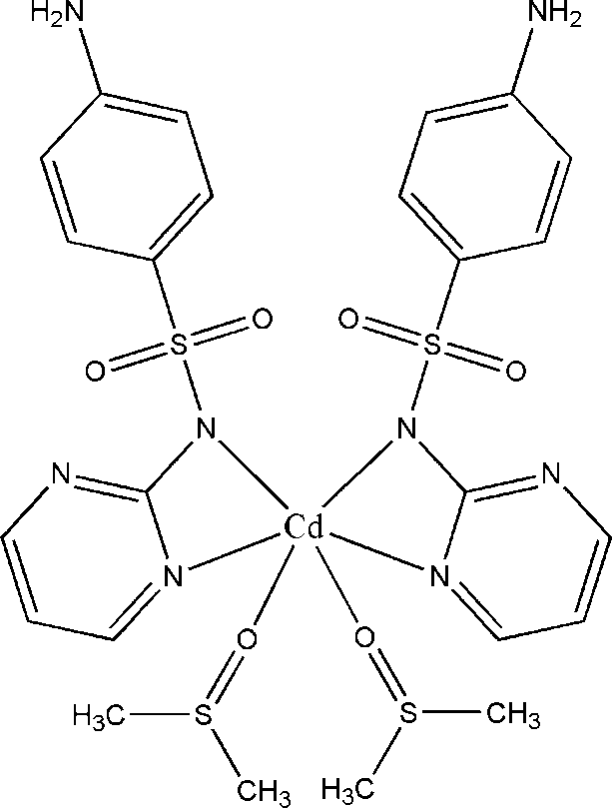


## Experimental

### 

#### Crystal data

[Cd(C_10_H_9_N_4_O_2_S)_2_(C_2_H_6_OS)_2_]
*M* *_r_* = 767.20
Orthorhombic, 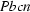

*a* = 16.9168 (5) Å
*b* = 15.2448 (3) Å
*c* = 11.8402 (3) Å
*V* = 3053.51 (13) Å^3^
*Z* = 4
Mo *K*α radiation
μ = 1.04 mm^−1^
*T* = 150 K
0.22 × 0.20 × 0.18 mm

#### Data collection

Nonius KappaCCD diffractometer
Absorption correction: multi-scan (Blessing, 1995 ▶) *T*_min_ = 0.803, *T*_max_ = 0.835
21431 measured reflections
3503 independent reflections
2545 reflections with *I* > 2σ(*I*)
*R*_int_ = 0.088

#### Refinement

*R*[*F* ^2^ > 2σ(*F* ^2^)] = 0.045
*wR*(*F* ^2^) = 0.102
*S* = 1.09
3503 reflections
235 parameters
30 restraints
H atoms treated by a mixture of independent and constrained refinement
Δρ_max_ = 0.74 e Å^−3^
Δρ_min_ = −0.78 e Å^−3^

### 

Data collection: *DENZO* (Otwinowski & Minor, 1997 ▶) and *COLLECT* (Hooft, 1998 ▶); cell refinement: *DENZO* and *COLLECT*; data reduction: *DENZO* and *COLLECT*; program(s) used to solve structure: *SHELXS97* (Sheldrick, 2008 ▶); program(s) used to refine structure: *SHELXL97* (Sheldrick, 2008 ▶); molecular graphics: *ORTEP-3 for Windows* (Farrugia, 1997 ▶); software used to prepare material for publication: *WinGX* (Farrugia, 1999 ▶).

## Supplementary Material

Crystal structure: contains datablocks I, global. DOI: 10.1107/S1600536811010816/tk2721sup1.cif

Structure factors: contains datablocks I. DOI: 10.1107/S1600536811010816/tk2721Isup2.hkl

Additional supplementary materials:  crystallographic information; 3D view; checkCIF report

## Figures and Tables

**Table 1:** Selected bond lengths (Å)

Cd1—O1	2.268 (3)
Cd1—N11	2.305 (3)
Cd1—N12	2.452 (3)

**Table 2:** Hydrogen-bond geometry (Å, °)

*D*—H⋯*A*	*D*—H	H⋯*A*	*D*⋯*A*	*D*—H⋯*A*
N14—H14*A*⋯N13^i^	0.94 (3)	2.30 (3)	3.168 (5)	152 (4)
N14—H14*B*⋯O11^ii^	0.95 (3)	2.02 (3)	2.967 (5)	173 (4)

Symmetry codes: (i) 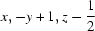; (ii) 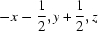.

**Fig. 1.**
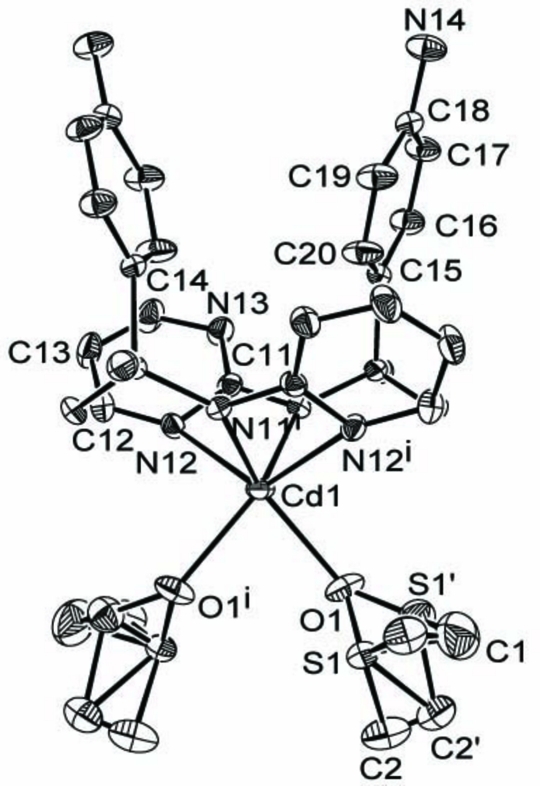
The molecular structure of (I), with atom labels and 50% probability displacement ellipsoids for non-H atoms. The S atom and methyl groups of dimethylsulfoxide are disordered over two sites, with occupancies of 0.715 (4) and 0.285 (4)

**Fig. 2.**
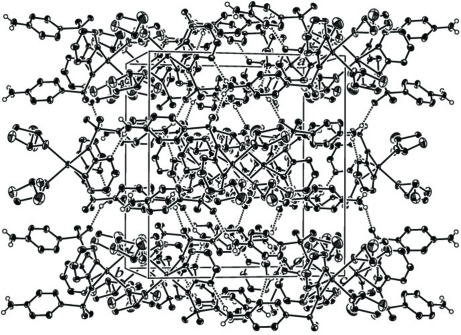
The packing of (I), viewed down the a-axis, showing one layer of molecules connected by N—H···N and N—H···O hydrogen bonds (dashed lines). H atoms not involved in hydrogen bonding have been omitted.

## supplementary crystallographic information

### Comment

In an attempt to prepare a complex of sulfadiazine with the cadmium(II) ion in methanol by the reaction of a cadmium salt and sulfadiazine followed by crystallization from dimethylsulfoxide, the compound isolated was shown by crystallography to have the formula [Cd(C_10_H_9_N_4_O_2_S)_2_](DMSO)_2_.

The six coordinate cadmium complex is trigonal prismatic, Fig. 1. The C18–N14 bond distance of 1.366 (5) Å and the C15–S11–N11–C11 torsion angle of 66.1 (3) ° are comparable to those observed in related structures (Heren *et al.*, 2006; Hossain & Amoroso, 2007). The Cd—O bond distance is shorter than the Cd—N bonds (Table 1). The dihedral angle between the aromatic rings of the anion of 88.65 (12) ° and this is greater than value of 71.10 (14) ° in the sulfadiazinate anion (Hossain & Amoroso, 2007). This is because in the latter the molecule is not bonded to a metal ion. The packing of (I) (Fig. 2) is stabilized by intermolecular N—H···N and N—H···O hydrogen bonds (Table 2) occurring between the anions in accord with a literature precedent (Paşaoğlu, *et al.*, 2008). This hydrogen bonding leads to layers in the *ab* plane, Fig. 2.

### Experimental

The sodium salt of sulfadiazine (5.446 g, 2 mmol) was dissolved in hot methanol (50 ml) and a methanol solution (10 ml) of (CH_3_COO)_2_Cd.2H_2_O (2.6647 g, 1 mmol) was added slowly with constant stirring on a hot plate. A white precipitate was formed and the mixture was stirred for a further 2 h. The precipitate was filtered off and dried over silica gel. It was then dissolved in dimethylsulfoxide solution (50 ml), Colourless blocks were filtered off and dried over silica gel.

### Refinement

The S atoms and methyl groups of dimethylsulfoxide were disordered. This was modelled with two different orientations and from refinement the site occupancies were 0.715 (4):0.285 (4). The H atoms were positioned geometrically and refined using in the riding model approximation, with C—H = 0.95–0.98 Å, and with *U*_iso_(H) = 1.2 (1.5 for methyl groups) times *U*_eq_(C). The N—H H atoms were located from a difference map and refined freely.

### Crystal data

**Table:**

[Cd(C_10_H_9_N_4_O_2_S)_2_(C_2_H_6_OS)_2_]	*F*(000) = 1560
*M**_r_* = 767.20	*D*_x_ = 1.669 Mg m^−^^3^
Orthorhombic, *P**b**c**n*	Mo *K*α radiation, λ = 0.71073 Å
Hall symbol: -P 2n 2ab	Cell parameters from 3503 reflections
*a* = 16.9168 (5) Å	θ = 2.9–27.5°
*b* = 15.2448 (3) Å	µ = 1.04 mm^−^^1^
*c* = 11.8402 (3) Å	*T* = 150 K
*V* = 3053.51 (13) Å^3^	Block, white
*Z* = 4	0.22 × 0.20 × 0.18 mm

### Data collection

**Table:**

Nonius KappaCCD diffractometer	3503 independent reflections
Radiation source: fine-focus sealed tube	2545 reflections with *I* > 2σ(*I*)
graphite	*R*_int_ = 0.088
ω scans	θ_max_ = 27.5°, θ_min_ = 3.2°
Absorption correction: multi-scan (Blessing, 1995)	*h* = −21→18
*T*_min_ = 0.803, *T*_max_ = 0.835	*k* = −19→19
21431 measured reflections	*l* = −15→15

### Refinement

**Table:**

Refinement on *F*^2^	Primary atom site location: structure-invariant direct methods
Least-squares matrix: full	Secondary atom site location: difference Fourier map
*R*[*F*^2^ > 2σ(*F*^2^)] = 0.045	Hydrogen site location: inferred from neighbouring sites
*wR*(*F*^2^) = 0.102	H atoms treated by a mixture of independent and constrained refinement
*S* = 1.09	*w* = 1/[σ^2^(*F*_o_^2^) + (0.0172*P*)^2^ + 6.2081*P*] where *P* = (*F*_o_^2^ + 2*F*_c_^2^)/3
3503 reflections	(Δ/σ)_max_ = 0.001
235 parameters	Δρ_max_ = 0.74 e Å^−^^3^
30 restraints	Δρ_min_ = −0.78 e Å^−^^3^

### Special details

**Table:**

Geometry. All e.s.d.'s (except the e.s.d. in the dihedral angle between two l.s. planes) are estimated using the full covariance matrix. The cell e.s.d.'s are taken into account individually in the estimation of e.s.d.'s in distances, angles and torsion angles; correlations between e.s.d.'s in cell parameters are only used when they are defined by crystal symmetry. An approximate (isotropic) treatment of cell e.s.d.'s is used for estimating e.s.d.'s involving l.s. planes.
Refinement. Refinement of *F*^2^ against ALL reflections. The weighted *R*-factor *wR* and goodness of fit *S* are based on *F*^2^, conventional *R*-factors *R* are based on *F*, with *F* set to zero for negative *F*^2^. The threshold expression of *F*^2^ > σ(*F*^2^) is used only for calculating *R*-factors(gt) *etc*. and is not relevant to the choice of reflections for refinement. *R*-factors based on *F*^2^ are statistically about twice as large as those based on *F*, and *R*- factors based on ALL data will be even larger.

### Fractional atomic coordinates and isotropic or equivalent isotropic displacement parameters (Å^2^)

**Table:**

	*x*	*y*	*z*	*U*_iso_*/*U*_eq_	Occ. (<1)
Cd1	0.0000	0.12725 (2)	0.2500	0.02037 (13)	
S11	−0.15957 (6)	0.26178 (6)	0.36236 (8)	0.0199 (2)	
O11	−0.20168 (16)	0.21509 (17)	0.2743 (2)	0.0270 (7)	
O12	−0.19685 (16)	0.26244 (17)	0.4719 (2)	0.0257 (6)	
N11	−0.07346 (19)	0.21814 (19)	0.3648 (3)	0.0198 (7)	
N12	0.05166 (19)	0.1969 (2)	0.4215 (3)	0.0199 (7)	
N13	−0.0247 (2)	0.3085 (2)	0.5150 (3)	0.0249 (8)	
N14	−0.1601 (2)	0.6222 (2)	0.1768 (3)	0.0303 (8)	
H14A	−0.129 (2)	0.630 (3)	0.111 (3)	0.049 (15)*	
H14B	−0.204 (2)	0.655 (3)	0.204 (4)	0.050 (15)*	
C11	−0.0156 (2)	0.2441 (2)	0.4378 (3)	0.0192 (8)	
C12	0.1152 (2)	0.2199 (3)	0.4811 (3)	0.0262 (9)	
H12	0.1637	0.1901	0.4685	0.031*	
C13	0.1122 (3)	0.2854 (3)	0.5601 (4)	0.0328 (11)	
H13	0.1575	0.3016	0.6026	0.039*	
C14	0.0402 (3)	0.3271 (3)	0.5751 (4)	0.0329 (11)	
H14	0.0367	0.3714	0.6313	0.039*	
C15	−0.1503 (2)	0.3716 (2)	0.3178 (3)	0.0206 (8)	
C16	−0.1968 (3)	0.4361 (3)	0.3654 (3)	0.0280 (10)	
H16	−0.2269	0.4232	0.4312	0.034*	
C17	−0.2005 (3)	0.5188 (3)	0.3195 (4)	0.0288 (10)	
H17	−0.2336	0.5619	0.3530	0.035*	
C18	−0.1556 (2)	0.5402 (2)	0.2229 (3)	0.0231 (9)	
C19	−0.1065 (3)	0.4757 (3)	0.1783 (4)	0.0312 (10)	
H19	−0.0739	0.4889	0.1152	0.037*	
C20	−0.1045 (3)	0.3927 (3)	0.2248 (4)	0.0332 (11)	
H20	−0.0711	0.3493	0.1924	0.040*	
O1	−0.08770 (19)	0.01537 (18)	0.2349 (2)	0.0364 (8)	
S1	−0.08063 (10)	−0.05478 (10)	0.14466 (12)	0.0277 (5)	0.715 (4)
S1'	−0.1400 (3)	−0.0149 (3)	0.1457 (3)	0.0378 (15)	0.285 (4)
C1	−0.1509 (6)	−0.0296 (6)	0.0397 (7)	0.068 (2)	0.715 (4)
H1A	−0.2036	−0.0265	0.0739	0.101*	0.715 (4)
H1B	−0.1504	−0.0753	−0.0185	0.101*	0.715 (4)
H1C	−0.1379	0.0271	0.0053	0.101*	0.715 (4)
C2	−0.1237 (6)	−0.1479 (5)	0.2028 (7)	0.051 (2)	0.715 (4)
H2A	−0.0896	−0.1710	0.2628	0.076*	0.715 (4)
H2B	−0.1304	−0.1925	0.1440	0.076*	0.715 (4)
H2C	−0.1754	−0.1327	0.2346	0.076*	0.715 (4)
C1'	−0.0900 (11)	−0.0263 (12)	0.0231 (14)	0.042 (4)	0.285 (4)
H1'1	−0.0843	0.0311	−0.0132	0.064*	0.285 (4)
H1'2	−0.1192	−0.0659	−0.0271	0.064*	0.285 (4)
H1'3	−0.0375	−0.0508	0.0384	0.064*	0.285 (4)
C2'	−0.1696 (10)	−0.1245 (11)	0.1724 (15)	0.040 (4)	0.285 (4)
H2'1	−0.1269	−0.1646	0.1512	0.060*	0.285 (4)
H2'2	−0.2169	−0.1380	0.1278	0.060*	0.285 (4)
H2'3	−0.1816	−0.1315	0.2529	0.060*	0.285 (4)

### Atomic displacement parameters (Å^2^)

**Table:**

	*U*^11^	*U*^22^	*U*^33^	*U*^12^	*U*^13^	*U*^23^
Cd1	0.0263 (2)	0.0129 (2)	0.0219 (2)	0.000	−0.00159 (18)	0.000
S11	0.0156 (5)	0.0151 (5)	0.0291 (5)	−0.0010 (4)	−0.0004 (4)	0.0004 (4)
O11	0.0217 (15)	0.0207 (15)	0.0386 (17)	−0.0055 (12)	−0.0096 (13)	−0.0029 (12)
O12	0.0208 (15)	0.0236 (15)	0.0328 (15)	0.0002 (12)	0.0050 (12)	0.0048 (12)
N11	0.0178 (17)	0.0148 (16)	0.0269 (17)	0.0027 (13)	−0.0023 (15)	−0.0019 (13)
N12	0.0162 (17)	0.0201 (17)	0.0234 (16)	0.0028 (14)	0.0008 (14)	0.0010 (13)
N13	0.0215 (19)	0.0261 (18)	0.0270 (18)	−0.0002 (15)	0.0013 (15)	−0.0092 (14)
N14	0.039 (2)	0.0210 (18)	0.0309 (19)	0.0042 (18)	0.0156 (18)	0.0058 (15)
C11	0.017 (2)	0.0179 (19)	0.0223 (19)	−0.0025 (16)	0.0031 (16)	0.0003 (15)
C12	0.018 (2)	0.033 (2)	0.028 (2)	0.0020 (18)	−0.0021 (18)	0.0006 (18)
C13	0.023 (2)	0.042 (3)	0.033 (2)	−0.004 (2)	−0.0073 (19)	−0.010 (2)
C14	0.028 (3)	0.038 (3)	0.033 (2)	−0.002 (2)	−0.005 (2)	−0.017 (2)
C15	0.018 (2)	0.0148 (19)	0.029 (2)	0.0010 (17)	−0.0021 (17)	0.0018 (15)
C16	0.032 (3)	0.022 (2)	0.030 (2)	0.0020 (19)	0.0095 (19)	0.0010 (17)
C17	0.030 (2)	0.019 (2)	0.038 (2)	0.0068 (19)	0.012 (2)	−0.0014 (17)
C18	0.024 (2)	0.0173 (19)	0.028 (2)	−0.0019 (17)	−0.0015 (18)	−0.0007 (15)
C19	0.036 (3)	0.020 (2)	0.038 (2)	−0.0013 (19)	0.020 (2)	0.0011 (18)
C20	0.037 (3)	0.016 (2)	0.047 (3)	0.0023 (19)	0.019 (2)	−0.0017 (18)
O1	0.048 (2)	0.0229 (15)	0.0381 (17)	−0.0132 (15)	−0.0010 (15)	−0.0059 (13)
S1	0.0303 (11)	0.0219 (8)	0.0310 (8)	−0.0015 (7)	−0.0025 (7)	−0.0043 (6)
S1'	0.040 (3)	0.038 (3)	0.036 (2)	−0.001 (2)	0.0012 (19)	−0.0055 (18)
C1	0.074 (4)	0.066 (4)	0.063 (4)	0.014 (4)	−0.024 (4)	−0.011 (3)
C2	0.071 (4)	0.031 (3)	0.050 (4)	−0.012 (3)	0.008 (3)	−0.003 (3)
C1'	0.051 (6)	0.039 (6)	0.037 (6)	−0.004 (5)	0.005 (5)	−0.002 (4)
C2'	0.041 (6)	0.036 (6)	0.043 (6)	−0.008 (5)	0.005 (5)	−0.006 (4)

### Geometric parameters (Å, °)

**Table:**

Cd1—O1	2.268 (3)	C16—H16	0.9500
Cd1—O1^i^	2.268 (3)	C17—C18	1.412 (6)
Cd1—N11^i^	2.305 (3)	C17—H17	0.9500
Cd1—N11	2.305 (3)	C18—C19	1.391 (6)
Cd1—N12	2.452 (3)	C19—C20	1.380 (6)
Cd1—N12^i^	2.452 (3)	C19—H19	0.9500
S11—O12	1.442 (3)	C20—H20	0.9500
S11—O11	1.449 (3)	O1—S1'	1.452 (5)
S11—N11	1.602 (3)	O1—S1	1.516 (3)
S11—C15	1.762 (4)	S1—C2	1.738 (7)
N11—C11	1.364 (5)	S1—C1	1.763 (8)
N12—C12	1.333 (5)	S1'—C1'	1.689 (16)
N12—C11	1.361 (5)	S1'—C2'	1.774 (18)
N13—C14	1.339 (5)	C1—H1A	0.9800
N13—C11	1.351 (5)	C1—H1B	0.9800
N14—C18	1.366 (5)	C1—H1C	0.9800
N14—H14A	0.94 (3)	C2—H2A	0.9800
N14—H14B	0.95 (3)	C2—H2B	0.9800
C12—C13	1.369 (6)	C2—H2C	0.9800
C12—H12	0.9500	C1'—H1'1	0.9800
C13—C14	1.385 (6)	C1'—H1'2	0.9800
C13—H13	0.9500	C1'—H1'3	0.9800
C14—H14	0.9500	C2'—H2'1	0.9800
C15—C16	1.381 (5)	C2'—H2'2	0.9800
C15—C20	1.385 (6)	C2'—H2'3	0.9800
C16—C17	1.373 (6)		
			
O1—Cd1—O1^i^	82.45 (16)	N13—C14—H14	118.1
O1—Cd1—N11^i^	139.32 (10)	C13—C14—H14	118.1
O1^i^—Cd1—N11^i^	98.40 (11)	C16—C15—C20	118.6 (4)
O1—Cd1—N11	98.40 (11)	C16—C15—S11	120.3 (3)
O1^i^—Cd1—N11	139.32 (10)	C20—C15—S11	120.6 (3)
N11^i^—Cd1—N11	106.09 (15)	C17—C16—C15	121.2 (4)
O1—Cd1—N12	128.62 (10)	C17—C16—H16	119.4
O1^i^—Cd1—N12	91.54 (11)	C15—C16—H16	119.4
N11^i^—Cd1—N12	92.06 (11)	C16—C17—C18	120.5 (4)
N11—Cd1—N12	56.20 (11)	C16—C17—H17	119.7
O1—Cd1—N12^i^	91.54 (11)	C18—C17—H17	119.7
O1^i^—Cd1—N12^i^	128.62 (10)	N14—C18—C19	121.9 (4)
N11^i^—Cd1—N12^i^	56.20 (11)	N14—C18—C17	120.3 (4)
N11—Cd1—N12^i^	92.06 (11)	C19—C18—C17	117.8 (4)
N12—Cd1—N12^i^	128.72 (14)	C20—C19—C18	120.7 (4)
O12—S11—O11	115.81 (17)	C20—C19—H19	119.6
O12—S11—N11	112.60 (17)	C18—C19—H19	119.6
O11—S11—N11	104.83 (17)	C19—C20—C15	121.1 (4)
O12—S11—C15	107.54 (17)	C19—C20—H20	119.4
O11—S11—C15	107.16 (17)	C15—C20—H20	119.4
N11—S11—C15	108.61 (17)	S1'—O1—S1	46.5 (2)
C11—N11—S11	122.9 (3)	S1'—O1—Cd1	134.0 (2)
C11—N11—Cd1	99.3 (2)	S1—O1—Cd1	122.32 (18)
S11—N11—Cd1	136.84 (18)	O1—S1—C2	105.3 (3)
C12—N12—C11	117.4 (3)	O1—S1—C1	106.8 (3)
C12—N12—Cd1	147.1 (3)	C2—S1—C1	100.1 (5)
C11—N12—Cd1	92.8 (2)	O1—S1'—C1'	110.7 (7)
C14—N13—C11	114.8 (3)	O1—S1'—C2'	110.0 (6)
C18—N14—H14A	114 (3)	C1'—S1'—C2'	101.4 (9)
C18—N14—H14B	114 (3)	S1'—C1'—H1'1	109.5
H14A—N14—H14B	130 (4)	S1'—C1'—H1'2	109.5
N13—C11—N12	125.1 (3)	H1'1—C1'—H1'2	109.5
N13—C11—N11	123.9 (3)	S1'—C1'—H1'3	109.5
N12—C11—N11	110.9 (3)	H1'1—C1'—H1'3	109.5
N12—C12—C13	121.6 (4)	H1'2—C1'—H1'3	109.5
N12—C12—H12	119.2	S1'—C2'—H2'1	109.5
C13—C12—H12	119.2	S1'—C2'—H2'2	109.5
C12—C13—C14	117.1 (4)	H2'1—C2'—H2'2	109.5
C12—C13—H13	121.5	S1'—C2'—H2'3	109.5
C14—C13—H13	121.5	H2'1—C2'—H2'3	109.5
N13—C14—C13	123.8 (4)	H2'2—C2'—H2'3	109.5
			
O12—S11—N11—C11	−52.9 (3)	N12—C12—C13—C14	−0.2 (7)
O11—S11—N11—C11	−179.7 (3)	C11—N13—C14—C13	0.9 (6)
C15—S11—N11—C11	66.1 (3)	C12—C13—C14—N13	−2.1 (7)
O12—S11—N11—Cd1	140.9 (2)	O12—S11—C15—C16	−20.1 (4)
O11—S11—N11—Cd1	14.2 (3)	O11—S11—C15—C16	105.1 (3)
C15—S11—N11—Cd1	−100.1 (3)	N11—S11—C15—C16	−142.2 (3)
O1—Cd1—N11—C11	137.0 (2)	O12—S11—C15—C20	168.7 (3)
O1^i^—Cd1—N11—C11	48.8 (3)	O11—S11—C15—C20	−66.1 (4)
N11^i^—Cd1—N11—C11	−75.9 (2)	N11—S11—C15—C20	46.6 (4)
N12—Cd1—N11—C11	5.6 (2)	C20—C15—C16—C17	2.6 (6)
N12^i^—Cd1—N11—C11	−131.2 (2)	S11—C15—C16—C17	−168.8 (3)
O1—Cd1—N11—S11	−54.8 (3)	C15—C16—C17—C18	−1.0 (7)
O1^i^—Cd1—N11—S11	−142.9 (2)	C16—C17—C18—N14	179.3 (4)
N11^i^—Cd1—N11—S11	92.4 (3)	C16—C17—C18—C19	−1.6 (6)
N12—Cd1—N11—S11	173.9 (3)	N14—C18—C19—C20	−178.4 (4)
N12^i^—Cd1—N11—S11	37.1 (3)	C17—C18—C19—C20	2.5 (6)
O1—Cd1—N12—C12	125.0 (5)	C18—C19—C20—C15	−0.9 (7)
O1^i^—Cd1—N12—C12	43.4 (5)	C16—C15—C20—C19	−1.7 (7)
N11^i^—Cd1—N12—C12	−55.0 (5)	S11—C15—C20—C19	169.7 (4)
N11—Cd1—N12—C12	−163.1 (5)	O1^i^—Cd1—O1—S1'	−115.6 (4)
N12^i^—Cd1—N12—C12	−101.8 (5)	N11^i^—Cd1—O1—S1'	−21.5 (4)
O1—Cd1—N12—C11	−77.5 (2)	N11—Cd1—O1—S1'	105.5 (4)
O1^i^—Cd1—N12—C11	−159.1 (2)	N12—Cd1—O1—S1'	158.5 (3)
N11^i^—Cd1—N12—C11	102.5 (2)	N12^i^—Cd1—O1—S1'	13.1 (4)
N11—Cd1—N12—C11	−5.6 (2)	O1^i^—Cd1—O1—S1	−57.01 (18)
N12^i^—Cd1—N12—C11	55.66 (19)	N11^i^—Cd1—O1—S1	37.1 (3)
C14—N13—C11—N12	2.6 (6)	N11—Cd1—O1—S1	164.1 (2)
C14—N13—C11—N11	−176.9 (4)	N12—Cd1—O1—S1	−142.92 (18)
C12—N12—C11—N13	−4.7 (6)	N12^i^—Cd1—O1—S1	71.8 (2)
Cd1—N12—C11—N13	−171.2 (3)	S1'—O1—S1—C2	−86.3 (4)
C12—N12—C11—N11	174.9 (3)	Cd1—O1—S1—C2	151.5 (4)
Cd1—N12—C11—N11	8.4 (3)	S1'—O1—S1—C1	19.5 (4)
S11—N11—C11—N13	0.0 (5)	Cd1—O1—S1—C1	−102.7 (4)
Cd1—N11—C11—N13	170.5 (3)	S1—O1—S1'—C1'	−45.6 (7)
S11—N11—C11—N12	−179.5 (3)	Cd1—O1—S1'—C1'	50.6 (8)
Cd1—N11—C11—N12	−9.1 (3)	S1—O1—S1'—C2'	65.6 (7)
C11—N12—C12—C13	3.3 (6)	Cd1—O1—S1'—C2'	161.8 (6)
Cd1—N12—C12—C13	157.7 (4)		

Symmetry codes: (i) −*x*, *y*, −*z*+1/2.

### Hydrogen-bond geometry (Å, °)

**Table:**

*D*—H···*A*	*D*—H	H···*A*	*D*···*A*	*D*—H···*A*
N14—H14A···N13^ii^	0.94 (3)	2.30 (3)	3.168 (5)	152 (4)
N14—H14B···O11^iii^	0.95 (3)	2.02 (3)	2.967 (5)	173 (4)

Symmetry codes: (ii) *x*, −*y*+1, *z*−1/2; (iii) −*x*−1/2, *y*+1/2, *z*.
